# Mobius Assembly: A versatile Golden-Gate framework towards universal DNA assembly

**DOI:** 10.1371/journal.pone.0189892

**Published:** 2018-01-02

**Authors:** Andreas I. Andreou, Naomi Nakayama

**Affiliations:** 1 SynthSys Centre for Synthetic and Systems Biology, University of Edinburgh, Edinburgh, United Kingdom; 2 Institute of Molecular Plant Sciences, University of Edinburgh, Edinburgh, United Kingdom; 3 Centre for Science at Extreme Condition, University of Edinburgh, Edinburgh, United Kingdom; Imperial College London, UNITED KINGDOM

## Abstract

Synthetic biology builds upon the foundation of engineering principles, prompting innovation and improvement in biotechnology via a design-build-test-learn cycle. A community-wide standard in DNA assembly would enable bio-molecular engineering at the levels of predictivity and universality in design and construction that are comparable to other engineering fields. Golden Gate Assembly technology, with its robust capability to unidirectionally assemble numerous DNA fragments in a one-tube reaction, has the potential to deliver a universal standard framework for DNA assembly. While current Golden Gate Assembly frameworks (e.g. MoClo and Golden Braid) render either high cloning capacity or vector toolkit simplicity, the technology can be made more versatile—simple, streamlined, and cost/labor-efficient, without compromising capacity. Here we report the development of a new Golden Gate Assembly framework named Mobius Assembly, which combines vector toolkit simplicity with high cloning capacity. It is based on a two-level, hierarchical approach and utilizes a low-frequency cutter to reduce domestication requirements. Mobius Assembly embraces the standard overhang designs designated by MoClo, Golden Braid, and Phytobricks and is largely compatible with already available Golden Gate part libraries. In addition, dropout cassettes encoding chromogenic proteins were implemented for cost-free visible cloning screening that color-code different cloning levels. As proofs of concept, we have successfully assembled up to 16 transcriptional units of various pigmentation genes in both operon and multigene arrangements. Taken together, Mobius Assembly delivers enhanced versatility and efficiency in DNA assembly, facilitating improved standardization and automation.

## Introduction

Synthetic biology is a fast expanding field at the interface of biology and engineering that facilitates predictive engineering of living organisms with novel functionalities [1]. Engineering principles, such as standardization, modularity, and simplicity, are implemented with the aim of reducing the unpredictability of complex and often non-linear living systems. Standardized DNA parts with consistent and well-characterized functionalities can be utilized just like man-made standardized parts (e.g. metric or imperial screws and optic boards or circuitry components) in mechanical and electric engineering. This will enable the implementation of the engineering 'design-construct-test' cycle in biology, ultimately allowing expansion of potential designs and efficient construction and testing, while encouraging automation and community-wide part exchange and resource establishment. To realize such universal standardization in synthetic biology, however, there remain fundamental challenges to consolidate part compatibility and to simplify and streamline the construction process.

In typical synthetic biology construction, modular DNA parts (e.g. promoters, coding sequences and terminators) are assembled to build molecular devices (e.g. functional transcriptional units), which can be combined further to assemble genetic modules (e.g. biosynthetic pathways). In addition, standardization sets rules on how these modular parts are designed and assembled [2]. The use of widely accepted standard parts and assembly methods facilitates exchangeability among users, allowing the reusability of available constructs, as well as automation of construction. Simple design would aid efficiency and versatility of molecular engineering.

DNA assembly thus is a pivotal technology in synthetic biology, since it materializes the construction of molecular modules, such as transcriptional units (TUs) and genetic circuits, from individual DNA parts, such as promoters, coding sequences and terminators [3]. DNA synthesis technologies are rapidly improving to provide affordable *ex vivo* synthesis of large DNA sequences; however, DNA assembly is and will be the predominant strategy for the assembly of DNA fragments larger than 1kb for the foreseeable future [4]. Currently, there are several DNA assembly methods used across the synthetic biology community (for an extensive review, see [5]). Those methods mainly fall into three categories: long-overlap assembly (e.g. Gibson Assembly [6]), site-specific recombination that exploits phage integrases (e.g. Gateway cloning [7]), and restriction endonuclease-based strategies. The endonuclease-based assembly methods are the most commonly used category that allows standardization when following a specific framework and set of rules.

One of the first attempts to standardize a restriction enzyme-based DNA assembly method was BioBricks, which was developed more than a decade ago [8]. The reusability and simplicity of BioBricks make them popular; for example they became the standard DNA assembly framework for the iGEM (international Genetic Engineered Machine) competition, which has played an instrumental role in nurturing new generations of synthetic biologists and synthetic biology tools and innovations (http://igem.org). There have been efforts to alleviate their drawbacks, such as the in-frame stop codon in the fusion scar and frequent need for ‘domestication’ (i.e. elimination of internal restriction sites) [9–11]. However, the pairwise nature of BioBrick assembly makes the construction of multipart systems time-consuming. Cloning with BioBricks can be labor-intensive, since the digestion and ligation take place in separate reactions.

Within a few years following BioBricks, a new generation of DNA assembly technology called Golden Gate Assembly was introduced [12,13]. This is based on Type IIS restriction endonucleases, which cleave double-stranded DNA outside their recognition sites. They leave a short single-stranded overhang, whose sequence can be defined by users. Golden Gate Assembly employs *Bsa*I restriction sites, which are eliminated during subcloning, allowing simultaneous digestion and ligation in a one-pot/tube reaction. Additionally, the use of distinct 4bp overlaps allows unidirectional, scarless cloning of multiple parts, since they are molecular screws and screw holes that are specifically designated to find each other. Nonetheless, the original Golden Gate Assembly framework lacked reusability, since the composite parts (e.g. TUs) could not be assembled further in multigene constructs. It also lacked standardization, since no community-wide rule was defined for the 4bp overhangs. Major breakthroughs came when the MoClo and Golden Braid variants of Golden Gate Assembly were developed to enable the hierarchical construction of multi-TUs and the full reusability of composite parts [14–17].

The Golden Braid 2.0 framework uses a simple pairwise approach where multipartite expansion is achieved by switching between two levels, α and Ω [15]. The standard parts feed the α and Ω levels, and the shift between the α and Ω levels doubles the number of TUs. The core vector toolkit of Golden Braid is comprised of only five plasmids. To date, the Golden Braid toolkit is mainly targeted at plant systems, although it can be made compatible with other chassis with a few modifications. An updated version of Golden Braid, G.B 3.0, comes with an online list of reusable standard parts providing the characterization data [18].

On the other hand, the MoClo framework uses a complex, yet high capacity vector toolkit to achieve parallel assembly [16,17]. The standard parts feed Level 1, which is comprised of seven plasmids. Assembly of up to six TUs takes Level 1 to Level 2 or to Level M/P, each of which involves seven vectors and a suite of End-linkers. The first direction employs seven Level 2 vectors and 21 End Linkers and can assemble up to six TUs per round [16]. The use of seven of the End Linkers results in non-modular constructs, and the cloning cannot further continue to the next level. The exploitation of the other fourteen End Linkers allows the augmentation of up to six TUs. The second direction uses two more levels of vectors, M and P levels, seven for each, each of which contains seven End Linkers [17]. Furthermore, up to six TUs are fused in a Level M vector, while switching between the M and P levels allows multigene augmentation. The MoClo toolkit was initially released for general eukaryotic expression [16], which was then adapted for plants [19], mammalian cells [20] and yeast [21,22], and it has just recently been extended for use in *Escherichia coli* [23–25] with modifications in the vectors and/or assembly standards. The mammalian MoClo (mMoClo) includes six parts positioning vectors to generate the standard parts, nine TU positioning vectors (Level 1), nine linker vectors and one destination vector (Level 2), which give the capacity of up to 9TU construct [20]. Instead of distributing a set of vectors, MoClo Yeast Toolkit provides parts to construct the vectors and uses the Assembly Connectors for the generation of multi-TUs [21]. With the five current Assembly Connectors in the toolkit, users can assemble up to 6TUs, but by constructing new Assembly Connectors they can expand the assembly capacity. The EcoFlex MoClo vector toolkit for *E*. *coli* contains two Level 0, six Level 1, six Level 2, two Level 3 vectors, and two Secondary Modules [24]. Level 2 can receive up to 5TUs from Level 1, and up to 20TUs can be assembled into Level 3. The CIDAR MoClo toolkit contains six Transcriptional Unit Vectors (DVK) and sixteen Basic Part/Device Vectors (DVA) which allows in two levels the generation of up to 4 part devices/circuits [25]. In all cases, MoClo systems tend toward more complex vector toolkits, sometimes with capped cloning capacity in some.

Assembly speed, toolkit and protocol simplicity, and cloning capacity make a DNA-assembly method attractive to users; however, the two most popular Golden Gate variants compromise at least one of them. Golden Braid sacrifices capacity of multi-gene cloning in favor of simplicity, while MoClo emphasizes cloning capacity and in so doing complicates its vector toolkit. A simple assembly method helps users assimilate and troubleshoot their cloning or vector toolkit problems; on the other hand, high capacity assembly methods are preferred, because they are time- and cost-effective. Furthermore, since both methods implement Type IIS restriction enzymes that are frequent cutters, they are burdened with heavy requirements for domestication, which is labor-intensive.

In order to address the tradeoffs and limitations of these current methods, ultimately to encourage universal standardization in synthetic biology construction, we developed Mobius Assembly, a new, highly versatile framework for hierarchical Golden Gate Assembly. Mobius Assembly embodies both simplicity and cloning capacity and thus allows exponential and theoretically unlimited augmentation of TUs. The two-level design, comprised of four Acceptor Vectors in each level and seven Auxiliary Plasmids, enables a quadruple assembly with a compact vector toolkit. Mobius Assembly also adopts the 4bp standard overhangs defined by MoClo and Golden Braid to promote the sharing of standard parts. Another new feature, the replacement of a frequent cutter with the rare cutter *Aar*I reduces domestication needs. Furthermore, the vectors are demarcated with specific visible markers for cloning screening. As a proof of concept, we have used Mobius Assembly to successfully reconstruct multi-gene biosynthetic clusters to produce protoviolaceinic acid and carotenoids. Additionally, to validate the capacity of the cloning system, we built a 16TU construct.

## Results and discussion

### Design features of the Mobius Assembly framework

**Fig 1:**
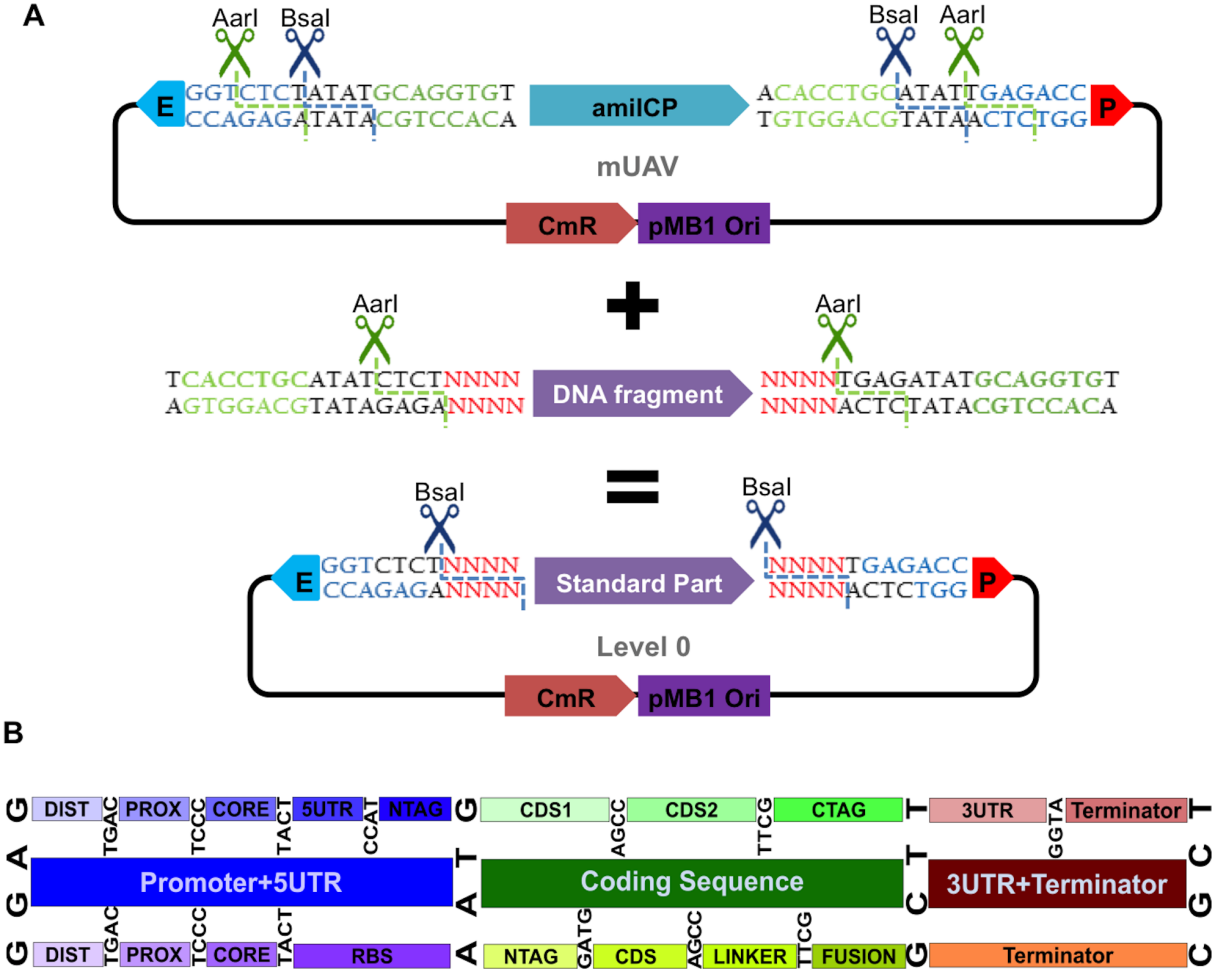
Mobius Assembly standard part generation. (A) Mobius Universal Acceptor Vector (mUAV) is the vector which converts and hosts DNA fragments as standard parts. mUAV is flanked by the Type IIS restriction enzymes *Bsa*I and *Aar*I and carries *amilCP* gene as visible cloning screening marker. The inserts are amplified with primers containing *Aar*I recognition sites, the fusion sites with the mUAV, and the standard overhangs, and they replace *amilCP* cassette in a Golden Gate reaction. The standard parts are released by *Bsa*I digestion. E: *Eco*RI; P: *Pst*I. (B) Mobius Assembly embraces the 4bp standard part overhangs defined by MoClo, Golden Braid, and Phytobricks, to facilitate part sharing. The middle row illustrates the standard overhangs for major functional parts (promoter, coding sequence, and terminator); the top row shows the recommended overhangs for eukaryotic sub-functional parts, while the bottom row indicates ones for the prokaryotic counterparts.

**Fig 2:**
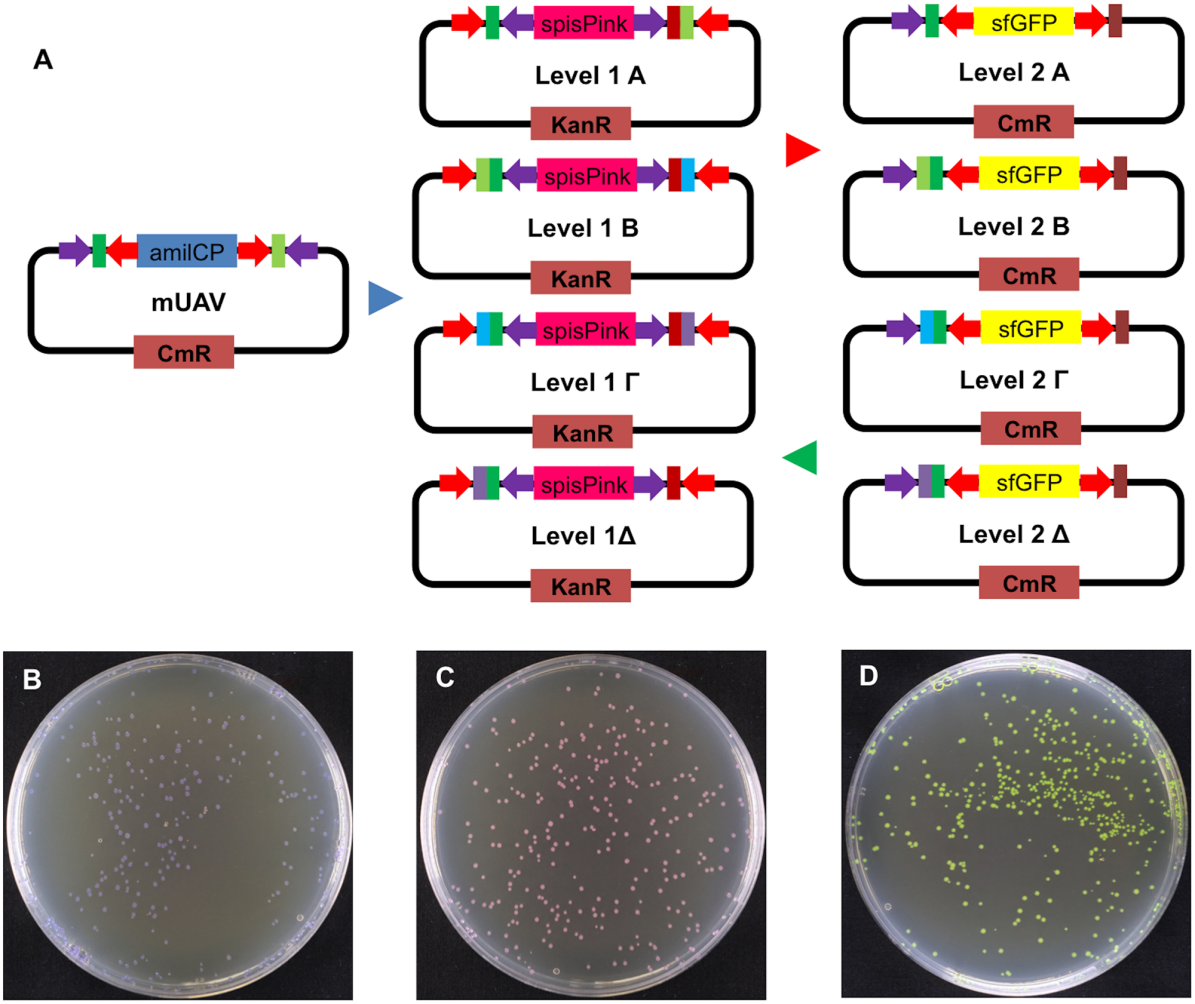
Mobius Assembly framework. (A) Mobius Assembly uses a two-level (Level 1 and 2) approach for transcriptional unit (TU) and multi-TU augmentation. Each level is comprised of four Acceptor Vectors. The four Level 1 Acceptor Vectors (A, B, Γ, and Δ) carry *spisPink gene* as the visible cloning screening marker and confer Kanamycin resistance. The four Level 2 Acceptor Vectors (A, B, Γ, and Δ) carry *sfGFP gene* as the visible cloning screening marker and confer Chloramphenicol resistance. The standard parts stored in mUAVs are released and fused in a Level 1 reaction to form a TU. Up to four Level 1 TUs can be fused in a Level 2 reaction to form a multi-TU cassette. Switching back and forth between Level 1 and 2 leads to further expansion of multi-TUs according to the geometric sequence: 1, 4, 16, 64,…. Red arrows denote *Aar*I restriction sites and Purple arrows *Bsa*I restriction sites. (B, C, and D) *E*. *coli* colonies carrying mUAV (B), Level Acceptor 1 Vector A (C), and Level 2 Acceptor Vector A (D), which respectively exhibit purple, magenta and yellow colour after overnight incubation. Successful assembly produces white colonies.

The Mobius Assembly framework commences at Level 0, which represents the standard part library. It uses the Mobius Universal Acceptor Vector (mUAV), to convert amplified PCR fragments into standard, exchangeable parts (Fig 1A). mUAV has a backbone derived from pSB1C3 and thus confers chloramphenicol resistance. We introduced the chromoprotein amilCP [26] as a visible cloning screening marker, which imbues a purple colour to the colonies (Fig 2B) (see below for the choice of the visible reporter genes). This negative screening marker is flanked by *Aar*I recognition sites. *Aar*I cuts through the *Bsa*I sequence, generating fusion sites (CTCT and TGAG) where a PCR fragment will be cloned in (Fig 1A). The insert should be amplified with a pair of primers each of which bear an *Aar*I restriction site, a fusion site that matches with the mUAV overhangs, and a 4bp standard overhang, from 5’ to 3’. *Aar*I digestion releases the *amilCP* gene, which is replaced by a standard part, resulting in a Level 0 plasmid. It should be noted that users can use any backbone in all levels of Mobius Assembly if the backbone we provide does not meet specific experimental requirements. Mobius Assembly cassettes are flanked with *Eco*RI and *Pst*I restriction sites, and the backbone can be swapped with a simple digestion/ligation step.

Mobius Assembly was designed such that the standard parts are released by *Bsa*I digestion, as in MoClo and Golden Braid, to facilitate exchangeability. At the same time, we introduced *Aar*I as a second restriction enzyme to address the domestication issue (Fig 1A). We opted for *Aar*I, because it is a rare cutter that recognizes the 7bp sequence CACCTGC(4/8)^ and leaves a 4bp overhang. Other Type IIS rare cutters leave 2 or 3bp overhangs or contain a large (e.g. 20bp) space between the recognition and cut sites. Golden Braid 2.0 [15] employs three restriction enzymes *Bsa*I, *Bsm*BI and *Btg*ZI, all of which recognize 6bp sequences. MoClo [16,17] also uses 6bp cutters *Bsa*I, *Bpi*I and sometimes *BsmB*I. The exchange of a 6bp cutter with *Aar*I thus theoretically drops domestication requirements by 58.3% compared to the systems using three 6bp cutters (e.g. Golden Braid 2.0) and by 37.5% to the systems using two 6bp cutters (e.g. CIDAR MoClo), while maintaining assembly efficiency and a 4bp overhang.

Mobius Assembly embraces the standard 4bp overhangs used by MoClo and Golden Braid. These specific sets of overhangs are becoming more common, being also adopted for Phytobricks, the newly emerging standard part collection for the iGEM registry (Fig 1B). Phytobricks were developed to propose a unifying design for universally exchangeable DNA parts [27]. Between MoClo and Golden Braid 2.0, there is partial compatibility of standard parts since they use the same sets of 4bp overhangs and use *Bsa*I for the TU assembly. Full compatibility is possible when a sequence is free from all restricted recognition sites used by each assembly framework. However, because the additional enzymes they require are frequent cutters, direct compatibility is limited. The scarcity of *Aar*I sites facilitates direct use of the available standard parts that have been generated by MoClo or Golden Braid, as well as Phytobricks, by reducing re-domestication requirements. We searched for the presence of *AarI* recognition sites through existing publically available standard parts compatible with Mobius Assembly, and we found none in great majority: for CIDAR MoClo only one out of 59 parts (cre_CD) contains an *AarI* site; from the MoClo Plant Parts Kit, four out of 95 contain *AarI* sites (pICSL80016, 43844, 75111, and 42222); in Golden Braid 2.0 Kit, 8 out of 56 parts need domestication for *AarI* (GB0082, 0145, 0096, 0208, 0575, 1041, 1079, and 0023); and all 20 Phytobricks in the iGEM distribution collection hold no *AarI* restriction sites. Therefore, Mobius Assembly is directly compatible with existing and future standard parts with other Golden Gate DNA assembly systems. Less domestication also renders Mobius Assembly more efficient for the generation of new standard parts.

To enable theoretically infinite assembly with a simple vector toolkit, we establish a two-level cloning framework that undergoes cycled, two-tier hierarchical augmentation, hence the name “Mobius Assembly”. The single TU assembly takes place in Level 1, and multi-TU assembly can be further continued by switching back and forth between Level 1 and Level 2 vectors (Fig 2A). Mobius Assembly enables the assembly or the addition of any number of composite parts as far as the vector or the chassis can handle.

**Fig 3:**
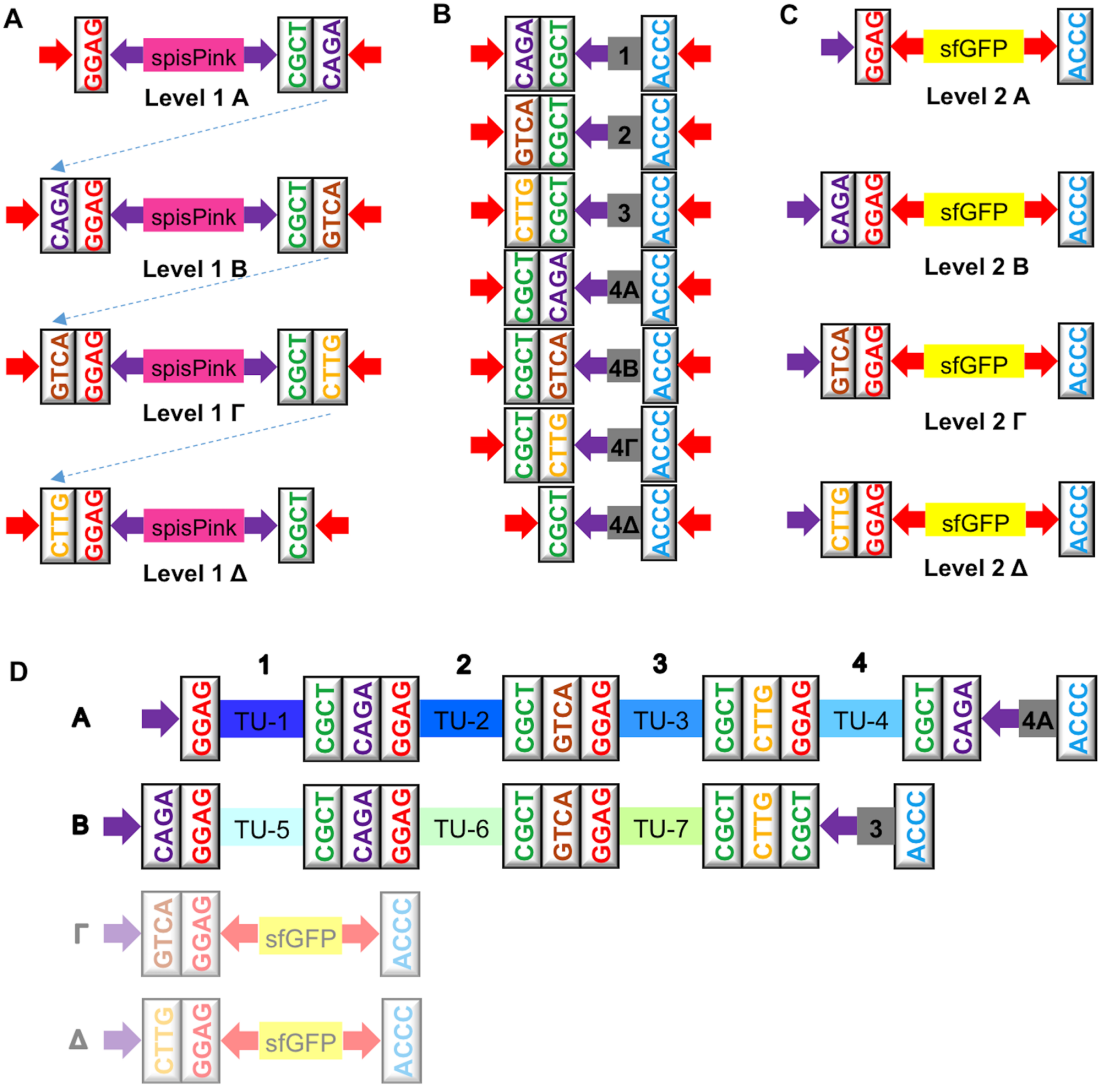
Mobius Assembly vector toolkit. (A) The overhangs of the four Level 1 Acceptor Vectors. *BsaI* digestion releases the *spisPink* gene upon digestion to expose GGAG and CGCT, between which a TU will be incorporated. Each type of vector has unique overhangs at the 3’ end which guides the assembly of up to four TUs in a Level 2 Acceptor Vector. (B) Seven Auxiliary Plasmids provide End-to-End linkers and Middle-to-End linkers to assist Level 2 cloning. (C) The overhangs of the four Level 2 Acceptor Vectors. Digestion with *Aar*I releases the *sfGFP* gene and exposes GGAG and ACCC, between which up to four TUs will be fused into with the assistance of an Auxiliary Plasmid. 4A, 4B, 4Γ and 4Δ End-to-End linkers provide 5’ and 3’ overhangs and the missing Level 2 overhang when four Level 1 TUs are fused. Middle-to-End linkers 1, 2, and 3 are used when one, two or three Level 1 cassettes are fused in Level 2. They provide 5’ and 3’ overhangs and the CGCT overhang necessary for the cloning back to Level 1. (D) An example of how the Auxiliary Plasmids are used. A 7-TU construct is generated by combining the four TUs in the Level 2 Acceptor Vector A and the remaining three TUs in Vector B in a Level 1 reaction. Auxiliary Plasmid 4A is used for the four TUs in Acceptor Vector A, and the Auxiliary Plasmid 3 for the three TUs in Vector B. Red arrows demarcate *Aar*I restriction sites and purple arrows *BsaI* restriction sites.

There are four Level 1 Acceptor Vectors, all of which are equipped with a kanamycin resistance gene, since the Mobius Assembly cassette is housed in the pSB1K3 backbone (Fig 2A). In Level 1 vectors, the *spisPink* chromoprotein gene [26] serves as a negative cloning screening marker, which colours the colonies pink (Fig 2C); it is released by *Bsa*I digestion and marks colonies with successfully assembled constructs as white. All Level 1 Acceptor Vectors contain the standard fusion sites at the 5’ (GGAG) and 3’ (CGCT) ends to house a (multi-)TU, plus the additional 4bp fusion sites for the cloning of up to four TUs in a Level 2 Acceptor Vector (Fig 3A).

Level 2 is comprised of four Acceptor Vectors, which have the pSB1C3 backbone that confers chloramphenicol resistance (Fig 2A). They all contain the *sfGFP* (superfolder GFP) gene [28] as a negative cloning screening marker, which makes the colonies yellow (Fig 2D); the screening marker is released by *Aar*I digestion, and successful assembly results in white colonies. Level 2 Acceptor Vectors have the same 5’ overhangs as Level 1 Acceptor Vectors and a common 3’ fusion site (ACCC), where the linkers from the Auxiliary Plasmids will anneal, providing the appropriate fusion sites to enable the assembly of up to four TUs in a Level 1 Acceptor Vector (Fig 3C).

In total, there are seven Auxiliary Plasmids that provide four End-to-End and three Middle-to-End 50bp linkers, which confer kanamycin resistance (Fig 3B). In the scenario where four Level 1 vectors are assembled into a Level 2 Acceptor Vector, an End-to-End Auxiliary Plasmid (4A, 4B, 4Γ, or 4Δ) is recruited to provide a linker containing three types of overhangs: i) the 5’ End overhang CGCT, which anneals to the 3’ overhang of Level 1 Vector Δ, ii) Level 2 End overhang depending on the type of Level 2 Acceptor Vector (4A = CAGA, 4B = GTCA, 4Γ = CTTG, or 4Δ = CGCT) used, and iii) the 3’ End overhang ACCC (Fig 3B and 3D). The Auxiliary Plasmid 4Δ is also used when eight or twelve TUs are assembled into B and Γ Acceptor Vectors, respectively. When less than four TUs are fused together, a Middle-to-End Auxiliary Plasmid (1, 2, or 3) is used to provide a linker containing three types of overhangs: i) 5’ end overhang depending on the number of TUs being combined (1 = CAGA, 2 = GTCA, or 3 = CTTG), ii) the overhang CGCT necessary to continue assembly back to Level 1, and iii) the 3’ end overhang ACCC (Fig 3B and 3D). Cloning from Level 2 to Level 1 does not require any Auxiliary Plasmids.

The design and workflow of Mobius Assembly framework caters to both capacity and simplicity. Having only four Acceptor Vectors in each level and seven Auxiliary Plasmids, Mobius Assembly vector toolkit is simple. Mobius Assembly framework elevates the assembly capability, in the manner descripted by the exponential geometric sequence a_n_ = a_1_r^n−^1 where *a1* = 1, *r* = 4 and n = 1, 2, 3, 4, and so on. Moreover, addition of new TU (single or multiple) in an already constructed multi-TU is possible by switching between the two cloning levels.

Since the protocol for Golden Gate Assembly with *Aar*I has not previously been optimized, we tested different ligases and buffers and chose to use T4 DNA ligase and T4 DNA ligase buffer in Level 2 reactions for the highest number of successful clones (S1 Fig and S1 Table). Several thermocycling conditions for restriction digestion and ligation were also tested, but for 4TU Level 2 assembly we did not detect clear differences in the cloning efficiency (S2 Fig and S2 Table).

The last feature we have introduced into Mobius Assembly is visible cloning screening by constitutively expressed chromogenic proteins, which replace Blue-White screening with the inducible lacZ operon. To identify effective visible markers, we screened eight chromoproteins (amilCP, amilGFP, spisPink, asPink, aeBlue, mRFP1, and tsPurple) and sfGFP (superfolder GFP), for strong and fast colour development during overnight incubation. Expression of these marker genes are controlled by the Anderson promoter J23106 and the rrnBT1-T7TE terminator. *amilCP*, *spisPink*, and *sfGFP*, which, after overnight incubation, develop strong purple, magenta, and yellow colours, respectively, (Fig 2B–2D), were selected as cloning screening markers for Level 0, 1, and 2. The strength and speed of colour development was strain-dependent. After overnight incubation expression for all the chromogenic genes was faster in TOP10 strain than in DH5α; while the colour was clearly identifiable in TOP10, in DH5α it was only possible after longer incubation (e.g. 24 hours). The difference in speed of colour development is probably due to differences in plasmid copy number, as we could extract higher concentrations of the plasmid from TOP10.

The benefits of cloning screening with chromogenic proteins are multifold. By eliminating the need for two expensive chemicals—IPTG and chromogenic substrate (X-gal)–the cloning screening becomes less costly. In addition, cloning chassis are no longer confined to the *E*. *coli* strains harboring the lacZΔM15 deletion mutation necessary for X-Gal screening. Furthermore, the use of distinct colours in each cloning level assists users in distinguishing between different cloning levels, and can be exploited by automated assembly platforms.

### Proof-of-concept experiments

To validate Mobius DNA Assembly in functionally reconstructing multigene constructs, we assembled genes involved in the violacein and carotenoid biosynthesis pathways as well as chromoprotein TUs. For the proof-of-concept experiment, pigmentation genes were chosen as their colour development facilitated the identification of the correctly assembled constructs with the naked eye.

**Fig 4:**
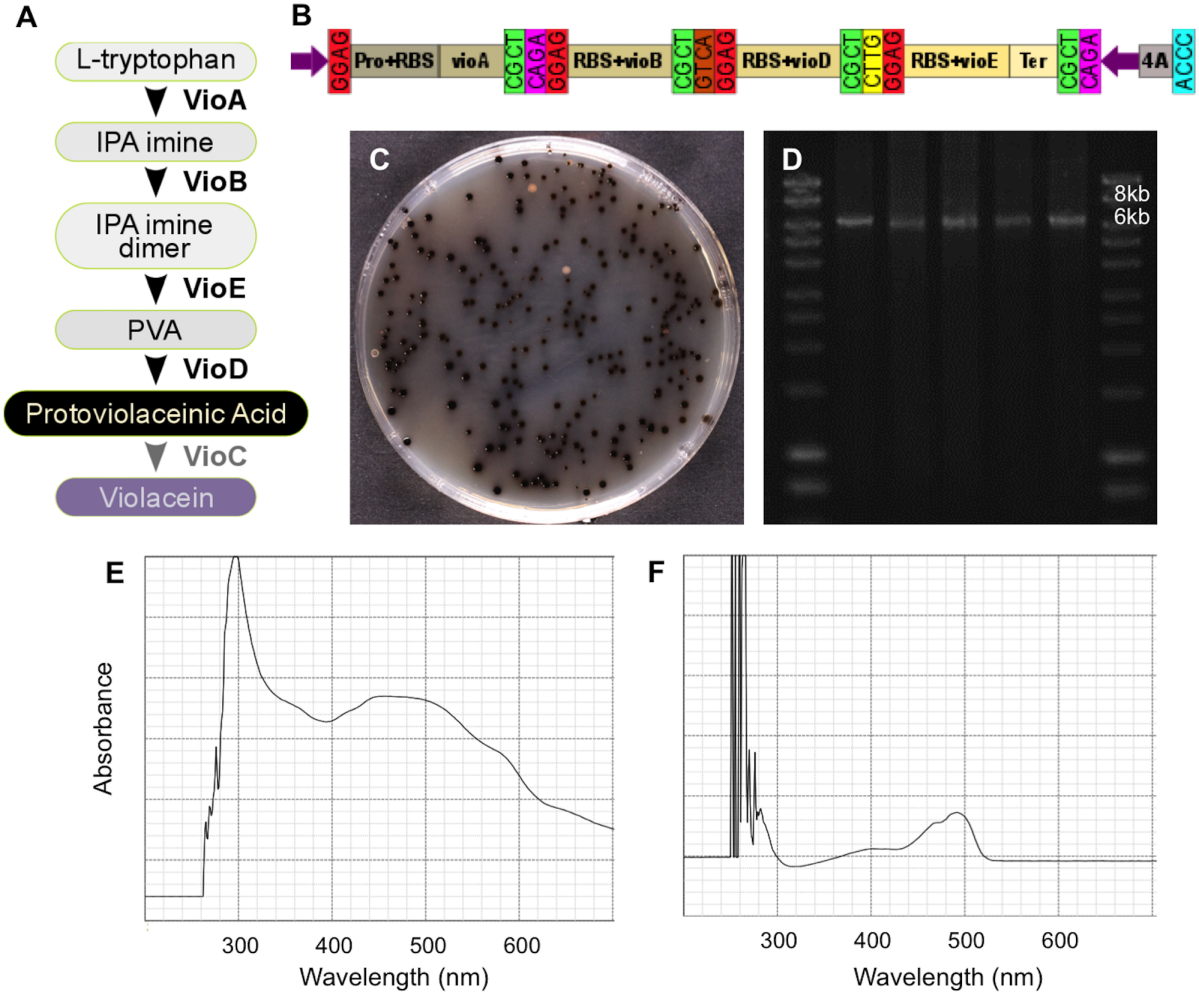
Reconstruction of the violacein biosynthesis operon. (A) Schematic of the violacein biosynthetic pathway showing enzymes mediating the conversion of the intermediates. (B) Diagram showing the assembly of *vioA*, *vioB*, *vioD* and *vioE* in a Level 2 Acceptor Vector A. (C) Cells transformed with the *vioABDE* operon form dark green to black colonies due to the production of protoviolaceinic acid. (D) Agarose gel electrophoresis of PCR products from five dark green/black colonies verified the correct size of the construct (6.5kb). (E) UV-Visible range spectrophotometry of the ethanol extract from a dark green colony showed a wide spectrum of absorbance indicative of protoviolaceinic acid. (F) Spectrophotometry of sfGFP extract from a colony without the recombinant plasmid. IPA: Indole-3-pyruvic acid; PVA: Protodeoxyviolaceinic acid.

As the first proof-of-concept experiment for Mobius Assembly, to reconstruct biosynthetic pathways organized into clusters sharing regulatory sequences, four genes from the violacein operon were re-assembled. Violacein is a bisindole pigment mainly produced in bacteria of the genus *Chromobacterium* [29]. The amino acid L-tryptophan, which is colourless within the visible range, is converted to the purple pigment violacein by the sequential activities of five enzymes co-localized in an operon: VioA, VioB, VioC, VioD and VioE (Fig 4A). VioA converts L-tryptophan into indole-3-pyruvic acid imine, which is then dimerized by VioB. VioE catalyzes the conversion of the dimer into protodeoxyviolaceinic acid, which is then converted to protoviolaceinic acid by VioD. The final product, violacein, results from conversion of protoviolaceinic acid via the action of VioC.

We first created the standard parts by PCR amplifying each of the four genes (*vioA*, *vioB*, *vioD* and *vioE*) using BBa_K598019 as the template and cloning it in the mUAV. *vioA* was amplified without a Ribosome Binding Site (RBS), while *vioB* and *vioD* were amplified with their RBS; for *vioE* a RBS was added via PCR primers. Next *vioA* coding sequence was fused to a weak promoter (Anderson Promoter J23103+B0034 RBS) in Level 1 Acceptor Vector A, *vioB* in Level 1 Acceptor Vector B, *vioD* in Level 1 Acceptor Vector Γ, and *vioE* with the rrnBT1-T7Te terminator in Level 1 Acceptor Vector Δ. Finally, the four genes were fused in Level 2 Acceptor Vector A (Fig 4B). The expression of the construct gave colonies with a deep green to black colour due to the production of protoviolaceinic acid, indicating successful reconstruction of the cluster (Fig 4C). Five colonies were selected for colony PCR, which resulted in products of the expected size (Fig 4D). The nature of the pigmentation was identified by spectrophotometry as spanning a wide range of emission wavelengths from UV to the visible spectrum, which is consistent with the dark blackish hues of the colonies (Fig 4E).

**Fig 5:**
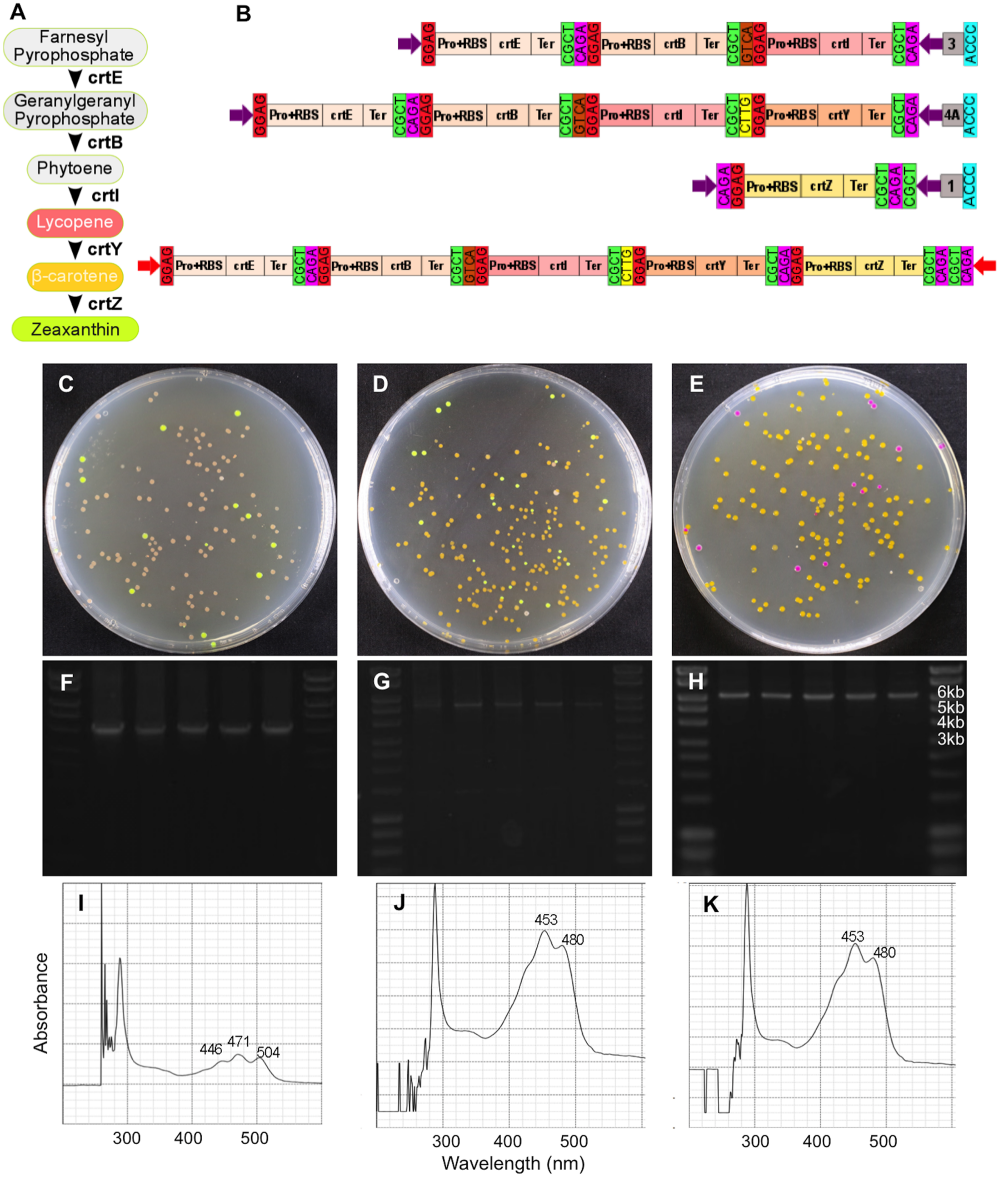
Reconstruction of the carotenoid biosynthesis transcriptional units. (A) A schematic of carotenoid biosynthetic pathway showing the enzymes mediating the production of zeaxanthin as the final product. (B) The multi-TU constructs made by Mobius Assembly to produce lycopene (*crtEBI*) and β-carotene (*crtEBIY*) (in Level 2 Acceptor Vectors) and for zeaxanthin (*crtEBIYZ*) by assembling *crtEBIY* and *crtZ* back in a Level 1 A Vector. Colonies producing lycopene are pink (C), β-carotene orange (D), and zeaxanthin yellow (E). Cells carrying intact Level 2 Vectors produced bright yellow colonies, and Level 1 Vectors pink colonies. Gel electrophoresis of the PCR from five colonies (pink, orange and yellow from each cloning, respectively) verified the correct size of the constructs; 4.3kb for lycopene (F), 5.7kb for β-carotene (G), and 6.7kb zeaxanthin (H). UV-Visible spectrophotometry showed expected peaks for lycopene (446nm, 472nm, and 503nm, I), β-carotene (450nm and 478nm, J) and zeaxanthin (450nm and 478nm, K).

To test the functional reconstruction of a biosynthetic pathway comprised of different TUs, rather than in an operon arrangement, five genes involved in carotenoid biosynthesis were assembled in three different combinations. Carotenoids are a group of omnipresent pigments produced by a diverse range of living organisms, including plants, algae, and microbes [30]. *E*. *coli* cannot naturally synthesize carotenoids; however, introduction of the carotenoid biosynthetic genes results in accumulation of specific variants [31]. The template used in this study is the carotenoid biosynthesis operon from *Pantoea ananatis* [32]. Farnesyl pyrophosphate (FPP), which is a precursor of several isoprenoid compounds, naturally exists in *E*. *coli* and is the substrate for carotenoid biosynthesis. Fig 5A depicts the carotenoid biosynthesis pathway. In the first step the enzyme geranylgeranyl pyrophosphate (GGPP) synthase encoded by the *crtE* gene takes isopentenyl pyrophosphate (IPP) along with farnesyl pyrophosphate (FPP) to generate GGPP; GGPP is then converted to a carotenoid intermediate, phytoene, via the action of phytoene synthase encoded by *crtB* gene. The first carotenoid, lycopene, exhibits a pink colour, and results from desaturation of phytoene by *crtI*. The lycopene cyclase from the *crtY* gene mediates conversion of lycopene to β-carotene, which is orange in colour. β-carotene is converted to yellow zeaxanthin by the enzyme beta-carotene hydroxylase encoded by *crtZ*.

Cloning of the carotenoid biosynthesis pathways proceeded as follows. Firstly, in Level 0, five genes involved in the carotenoid biosynthesis, *crtE*, *crtB*, *crtI*, *crtY* and *crtZ*, were cloned into the mUAV. Next we assembled them into TUs (promoter+RBS:coding_sequence:terminator), each of which included the terminator rrnBT1-T7Te. J23110+B0034:*crtE* and J23103+B0034:*crtZ* were cloned in Level 1 Acceptor Vector A and J23103+B0034:*crtB*, J23103+B0034:*crtI*, J23103+B0034:*crtY* in Level 1 Acceptor Vectors B, Γ, and Δ, respectively. The weak promoter J23103 was chosen after we observed that strong overexpression of these carotenoid biosynthesis genes was lethal to the cells (data not shown). To synthesize lycopene, three TUs *crtE*, *crtB*, and *crtI* were assembled in a Level 2 Acceptor Vector A (Fig 5B), and successfully constructed cassettes resulted in pink-coloured colonies (Fig 5C). For biosynthesis of β-carotene, four TUs *crtE*, *crtB*, *crtI*, and *crtY* were assembled, resulting in orange coloured colonies (Fig 5D). To form the final expression cassette TU *crtZ* was cloned in Level 2 Acceptor Vector B, which was fused with *crtEBIY* in a second assembly step back to Level 1 Acceptor Vector A. This five TU construct led to colonies with a yellow colour, consistent with zeaxanthin accumulation (Fig 5E). The size of the constructs was verified by colony PCR amplification of the insert (Fig 5F, 5G and 5H). The identity of the carotenoid variants produced by each construct was verified using spectrophotometry; the expected emission peaks [33] were observed for lycopene (Fig 5I), β-carotene (Fig 5J) and zeaxanthin (Fig 5K) extracts.

Lastly, we tested the hierarchical assembly capacity of Mobius Assembly by assembling a 16TU construct, which is 18.2kb in size (20.4kb with the vector). To create this high-level multi-TU construct, carotenoid, chromoprotein, and violacein TU modules were combined. The chromoproteins for this experiment were selected such that the individual colours would be detectable, even in combination. Eight chromoprotein genes were cloned into Level 1 Acceptor Vectors, each of which was combined with the weak promoter J23103+B0034 and weak transcription terminator for the *E*.*coli* RNA polymerase, T7Te. We chose to have expression of each gene low to avoid any possible toxicity due to over production of pigments and/or competition for transcription/translation machinery [34]. The chromoprotein TUs were grouped into two categories of four genes each, according to their colour ranges—yellow (*scOrange*, *amilGFP*, *amajLime*, *fwYellow*) and pink (*tsPurple*, *efforRed*, *asCP*, *mRFP1*)–and they were respectively assembled in Level 1 Acceptor Vectors A, B, Γ, and Δ.

**Fig 6:**
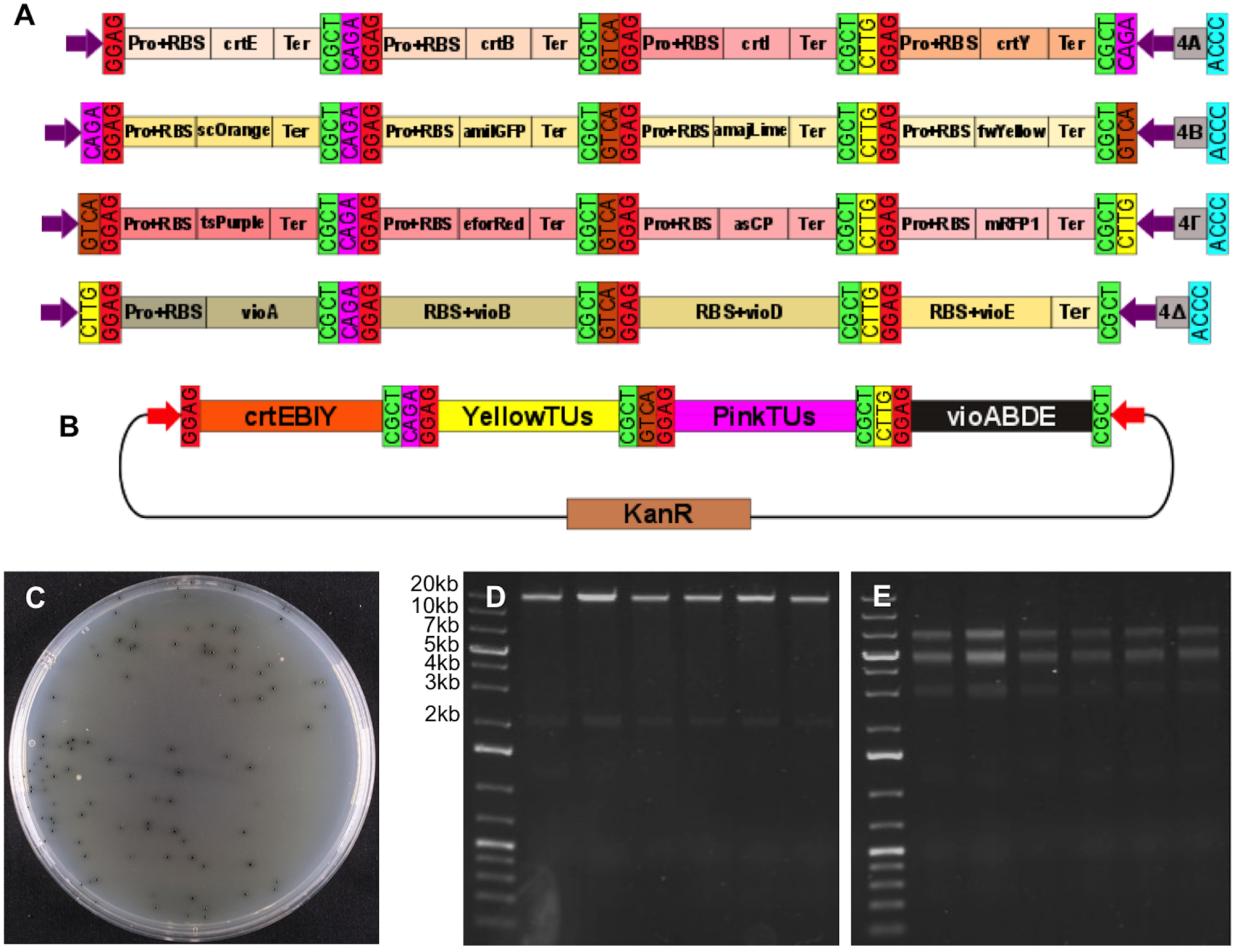
Proof-of-concept assembly of 16TU construct. (A) A schematic showing the four intermediate Level 2 constructs for the assembly of the 16-TU construct. The carotenoid biosynthesis genes *crtE*, *crtB*, *crtI*, and *crtY* assembled in the Vector A, the yellow chromoprotein genes *scOrange*, *amilGFP*, *amajLime*, and *fwYellow* in the Vector B, the pink chromoprotein genes *tsPurple*, *eforRed*, *spisPink*, and *mRFP1* in the Vector Γ, and the violacein biosynthesis genes *vioA*, *vioB*, *vioD* and *vioE* in the Vector Δ. (B) A schematic of the 16TU construct derived from the assembly of the four Level 2 cassettes, each containing 4-TUs, in the Level 1 Acceptor Vector A. (C) Cells transformed with the successfully assembled 16TU construct grew into black colonies due to predominant colouring by protoviolaceinic acid. (D) Gel electrophoresis of six plasmids (isolated from the black colonies) digested with *PstI* and *EcoRI* resulting in bands of expected sizes—18.2kb for the insert and 2.2kb for the vector. (E) The same plasmids were digested with *PstI* and *AleI* resulting in the bands of expected sizes—7.1kb, 5.1 and 4.9kb (appear merged on the gel), and 3.2kb.

The single chromoprotein TUs were then fused in Level 2 Acceptor Vectors to form 4TU constructs. More specifically, the yellow group was assembled in Level 2 Acceptor Vector B, and the pink group in Γ (Fig 6A). The successful constructs were identified firstly by their displayed composite colony colour (yellowish and pink) and secondly by colony PCR of the insert. The violacein operon was reconstructed as described above, but in Level 2 Acceptor Vector Δ. In addition, the carotenoid biosynthesis module *crtEBIY*, as described above, was used in Level 2 Acceptor Vector A. In the final assembly step, the four Level 2 Vectors were fused back to Level 1 Acceptor Vector A to generate the 16TU construct (Fig 6B). Again, the correct assemblies were distinguished by the black colour of the colonies due to dominant pigmentation by protoviolaceinic acid (Fig 6C). Six colonies were selected for DNA plasmid isolation and double restriction digestion with *EcoRI* and *PstI* or *PstI* and *AleI*, which resulted in the anticipated patterns of DNA bands (Fig 6D and 6E). The presence of all the 16 TUs in the final construct was further verified by Sanger sequencing.

## Conclusions

We have developed a simple yet versatile new Golden Gate Assembly framework, which was named Mobius Assembly for its (theoretically) unlimited cloning scheme that shifts between two tiers. Mobius Assembly builds upon the increasingly popular two current Golden Gate Assembly frameworks, MoClo and GB2.0, combining their strengths of comprising a simple vector toolkit, while conferring high cloning capacity. Mobius Assembly is equipped with visible cloning screening based on chromogenic proteins that demarcate different levels with distinct colors—a feature which can be used also as a security measure to ensure correct cloning steps in automated or manual cloning. We also opted for a low-frequency cutter *AarI* to reduce the domestication needs. The assembly was highly efficient; in all reactions we made, cloning was successful in almost all colonies tested (a few exceptions were found when similar genes were combined, having undergone recombination events). Multiple genes were functionally cloned in both operon and individual transcriptional unit arrangements, and thus Mobius Assembly is compatible with bioengineering of prokaryotic and eukaryotic systems. The streamlined DNA assembly enhances the efficiency, and user-friendliness of molecular cloning using Golden Gate Assembly, and we hope that Mobius Assembly will contribute to the establishment of universal standards in synthetic biology constructions. To this end, Mobius Assembly embraced the specific sets of overhangs that were defined by MoClo, GB2.0, and Phytobricks and are shared among a growing number of synthetic biologists using Golden Gate DNA assembly.

## Materials and methods

### Cell culturing and plasmid preparation

*E*. *coli* cells (DH5α or TOP10) were grown in 5 ml LB growth medium supplemented with appropriate antibiotics for overnight incubation at 37°C, 230 rpm. Plasmids were isolated using either Monarch (NEB) or PureYield (Promega) Plasmid Miniprep. The Inoue Method [35] was used to prepare home-made ultra-competent cells of DH5α (NEB) or TOP10 (Thermo Fisher Scientific), which were transformed with the constructs as follows: 5 μl of the DNA solution was incubated with 50 μl of the competent cells on ice for 10 min, followed by a heat shock at 42°C for 40 s and re-cooled on ice for 10 min. SOC medium (400 μl) was added, and after 1 hr incubation at 37°C, 200 rpm, 50 μl of the cell suspension was plated on LB agar plates with antibiotic selection. The plates were incubated overnight at 37°C.

### Part and vector generation

pSB1C3 (iGEM DNA distribution) was used as the backbone for mUAV and Level 2 Acceptor Vectors, while pSB1K3 (iGEM DNA distribution) was used for the construction of Level 1 Acceptor Vectors. In the preliminary system, pCR8 (Thermo Fisher Scientific) was used as the backbone, and some of our part standards are in the pCR8-based mUAV. Bacterial promoters and terminators, as well as the genes for the carotenoid and violacein biosynthetic pathways, were cloned from the iGEM DNA distribution kit. Chromoproteins were kind gifts from the Uppsala iGEM Association. All the standard parts were domesticated for *Aar*I and *Bsa*I when necessary and were cloned into mUAV.

To generate the Mobius Assembly TU cassettes for the vector toolkit, J23106 promoter, a chromoprotein gene (amilCP for mUAV, spisPink for Level 1 Acceptor Vectors, and sfGFP for Level 2 Acceptor Vectors), and the rrnBT1-T7Te terminator were combined using Golden Gate assembly. They were then amplified using Q5 DNA polymerase (NEB) with primers bearing *EcoRI*, *AarI*, *BsaI*, and *PstI* restriction sites and 4 bp overhangs. Subsequently the pSB1C3/pSB1K3 backbones and the Golden Gate cassettes were digested with EcoRI-HF and PstI-HF (NEB) for 20 min at 37°C followed by purification with PCR Clean-up kit (Macherey Nagel). Ligation was mediated by T7 DNA ligase (NEB) to construct mUAV, Level 1 and Level 2 Acceptor Vectors. To construct the 50 bp linkers in the Auxiliary Plasmids, a short sequence was PCR amplified from *scOrange* gene using primers that contained the appropriate overhangs and the *Aar*I and *Bsa*I recognition sites. The PCR products were purified and then digested for 2 hrs at 37°C with *Aar*I (Thermo Fisher Scientific). A Level 1 Acceptor Vector A was also digested with *AarI* and ligated to the Auxiliary Plasmid cassettes with 1 μl T7 DNA ligase (NEB) for 20min at RT. The constructs were verified by Sanger sequencing (GATC-Biotech or Edinburgh Genomics). The full sequences of the 16 Mobius Assembly toolkit vectors have been deposited to GenBank, and these vectors are publicly distributed from Addgene; their GenBank and Addgene IDs are listed in S3 Table.

### Golden gate assembly

The DNA assembly was carried out in 10 μl reaction comprised of ~50 ng Acceptor Vector and twice as many molar of the insert parts, in addition to 1 μl 1 mg/ml BSA (NEB), 1 μl T4 DNA ligase buffer (NEB or Thermo Fisher Scientific), 0.5 μl *Aar*I (Thermo Fisher Scientific) for cloning in mUAV and Level 2 Acceptor Vectors or *Eco*31I (*Bsa*I) (Thermo Fisher Scientific) for cloning in Level 1 Acceptor Vectors, and 0.5 μl T4 DNA ligase (NEB or Thermo Fisher Scientific). For reactions with *Aar*I, extra 0.2 μl 50x oligos (0.025 mM) of the enzyme recognition sites were added. The one-tube reaction was incubated in a thermocycler for five times cycles of (37°C for 5 min, 16°C for 10 min) followed by 5 min digestion at 37°C and 5 min deactivation at 80°C. For the assembly of the 16 TU construct, the reaction was set in 20 μl with double amount of buffers and enzymes and the thermocycling conditions were altered to: 40 cycles of (37°C for 2.5 min, 16°C for 5 min) followed by 5 min digestion at 37°C and 5 min deactivation at 80°C. The sequences of all the primers and the resulting plasmids have been deposited to the public database Edinburgh Datashare (http://dx.doi.org/10.7488/ds/2261).

### Pigment spectrophotometry

Lycopene, β-carotene, zeaxanthin, and protoviolaceinic acid were extracted from 100 ml overnight cultures in LB. Cells were harvested by centrifugation at 2500 x g for 10 min and then resuspended in 2 ml 96% ethanol and 2 min vortexing for cell lysis. The lysate was then centrifuged for 5 min at 14000 x g, and the supernatant was filtered through a 0.22 μm filter (Milipore) and used for spectrophotometry for the UV-visible range wavelengths (200–780 nm) using Biowave II (Montreal-biotech).

## Supporting information

S1 Fig Optimization of level 2 assembly reagents. Level 2 cloning was optimized for the different reaction buffers and DNA ligases. The chromoprotein TUs tsPurple, amilGFP, asCP, and aeBlue were combined in Level 2 Acceptor Vectors, and the cells with successfully assembled constructs grew into blue colonies. Different sets of reagents tested were: (A) Buffer AarI + T4 DNA ligase, (B) 2x Tango Buffer + T4 DNA ligase, (C) T4 DNA Ligase Buffer + T4 DNA ligase, (D) Buffer AarI + T7 DNA ligase, (E) 2x Tango Buffer T7 DNA ligase, (F) T4 DNA Ligase Buffer + T7 DNA ligase. T4 DNA Ligase Buffer + T4 DNA ligase combination resulted in the optimum assembly efficiency.
(PDF)

S2 Fig Optimization of level 2 assembly reaction conditions. Level 2 cloning was optimized for the different thermocycling conditions used for the assembly reactions. The chromoprotein TUs tsPurple, eforRed, asCP and mRFP1 were cloned into Level 2 Acceptor Vectors under different thermocycling conditions (digestion/ligation). Cells with successfully assembled constructs grew into pink colonies.
(PDF)

S1 Table Colony counts for the level 2 assembly reagents. (PDF)

S2 Table Colony counts for the level 2 assembly optimization. (PDF)

S3 Table Vector toolkit: Features and database and repository IDs. (PDF)

## References

[1] Andrianantoandro E, Basu S, Karig DK, Weiss R. Synthetic biology: new engineering rules for an emerging discipline. Mol Syst Biol. 2006;2 Available: http://msb.embopress.org/content/2/1/2006.0028.abstract 10.1038/msb4100073 PMC1681505 16738572

[2] Arkin A. Setting the standard in synthetic biology. Nat Biotechnol. 2008;26: 771–4. doi: 10.1038/nbt0708-771 18612298 10.1038/nbt0708-771

[3] Ellis T, Adie T, Baldwin GS. DNA assembly for synthetic biology: from parts to pathways and beyond. Integr Biol (Camb). 2011;3: 109–118. doi: 10.1039/c0ib00070a 21246151 10.1039/c0ib00070a

[4] Hughes RA, Ellington AD. Synthetic DNA Synthesis and Assembly : Putting the Synthetic in Synthetic Biology. 2017; doi: 10.1101/CSHPERSPECT.A023812 28049645 10.1101/cshperspect.a023812 PMC5204324

[5] Casini A, Storch M, Baldwin GS, Ellis T. Bricks and blueprints: methods and standards for DNA assembly. Nat Rev Mol Cell Biol. Nature Publishing Group; 2015;16: 568–576. doi: 10.1038/nrm4014 26081612 10.1038/nrm4014

[6] Gibson DG, Young L, Chuang R-Y, Venter JC, Hutchison CA, Smith HO. Enzymatic assembly of DNA molecules up to several hundred kilobases. Nat Meth. Nature Publishing Group; 2009;6: 343–345. Available: http://dx.doi.org/10.1038/nmeth.1318 10.1038/nmeth.1318 19363495

[7] Hartley JL, Temple GF, Brasch MA. DNA Cloning Using In Vitro Site-Specific Recombination. Genome Res. 2000;10: 1788–1795. 11076863 10.1101/gr.143000 PMC310948

[8] Shetty RP, Endy D, Knight TF. Engineering BioBrick vectors from BioBrick parts. J Biol Eng. 2008;2: 5 doi: 10.1186/1754-1611-2-5 18410688 10.1186/1754-1611-2-5 PMC2373286

[9] Anderson Jc, Dueber JE, Leguia M, Wu GC, Goler JA, Arkin AP, et al BglBricks: A flexible standard for biological part assembly. J Biol Eng. 2010;4: 1 doi: 10.1186/1754-1611-4-1 20205762 10.1186/1754-1611-4-1 PMC2822740

[10] Liu JK, Chen WH, Ren SX, Zhao GP, Wang J. IBrick: A new standard for iterative assembly of biological parts with homing endonucleases. PLoS One. 2014;9: 1–10. doi: 10.1371/journal.pone.0110852 25329380 10.1371/journal.pone.0110852 PMC4203835

[11] Li S, Zhao G, Wang J. C-Brick: A New Standard for Assembly of Biological Parts using Cpf1. ACS Synth Biol. 2016; acssynbio.6b00114. doi: 10.1021/acssynbio.6b00114 27294364 10.1021/acssynbio.6b00114

[12] Engler C, Kandzia R, Marillonnet S. A one pot, one step, precision cloning method with high throughput capability. PLoS One. 2008;3 doi: 10.1371/journal.pone.0003647 18985154 10.1371/journal.pone.0003647 PMC2574415

[13] Engler C, Gruetzner R, Kandzia R, Marillonnet S. Golden gate shuffling: a one-pot DNA shuffling method based on type IIs restriction enzymes. PLoS One. 2009;4: e5553 doi: 10.1371/journal.pone.0005553 19436741 10.1371/journal.pone.0005553 PMC2677662

[14] Sarrion-Perdigones A, Falconi EE, Zandalinas SI, Juárez P, Fernández-del-Carmen A, Granell A, et al GoldenBraid: An Iterative Cloning System for Standardized Assembly of Reusable Genetic Modules. PLoS One. Public Library of Science; 2011;6: e21622 Available: https://doi.org/10.1371/journal.pone.0021622 10.1371/journal.pone.0021622 PMC3131274 21750718

[15] Sarrion-Perdigones A, Vazquez-Vilar M, Palaci J, Castelijns B, Forment J, Ziarsolo P, et al GoldenBraid 2.0: A Comprehensive DNA Assembly Framework for Plant Synthetic Biology. Plant Physiol. 2013;162: 1618–1631. doi: 10.1104/pp.113.217661 23669743 10.1104/pp.113.217661 PMC3707536

[16] Weber E, Engler C, Gruetzner R, Werner S, Marillonnet S. A Modular Cloning System for Standardized Assembly of Multigene Constructs. PLoS One. 2011;6: e16765 doi: 10.1371/journal.pone.0016765 21364738 10.1371/journal.pone.0016765 PMC3041749

[17] Werner S, Engler C, Weber E, Gruetzner R, Marillonnet S. Fast track assembly of multigene constructs using golden gate cloning and the MoClo system. Bioeng Bugs. 2012;3: 38–43. doi: 10.1371/journal.pone.0016765 22126803 10.4161/bbug.3.1.18223

[18] Vazquez-Vilar M, Quijano-Rubio A, Fernandez-del-Carmen A, Sarrion-Perdigones A, Ochoa-Fernandez R, Ziarsolo P, et al GB3.0: a platform for plant bio-design that connects functional DNA elements with associated biological data. Nucleic Acids Res. 2017;45: gkw1326 doi: 10.1093/nar/gkw1326 28053117 10.1093/nar/gkw1326 PMC5389719

[19] Engler C, Youles M, Gruetzner R, Ehnert TM, Werner S, Jones JDG, et al A Golden Gate modular cloning toolbox for plants. ACS Synth Biol. 2014;3: 839–843. doi: 10.1021/sb4001504 24933124 10.1021/sb4001504

[20] Duportet X, Wroblewska L, Guye P, Li Y, Eyquem J, Rieders J, et al A platform for rapid prototyping of synthetic gene networks in mammalian cells. Nucleic Acids Res. 2014;42: 13440–13451. doi: 10.1093/nar/gku1082 25378321 10.1093/nar/gku1082 PMC4245948

[21] Lee ME, DeLoache WC, Cervantes B, Dueber JE. A Highly Characterized Yeast Toolkit for Modular, Multipart Assembly. ACS Synth Biol. 2015;4: 975–986. doi: 10.1021/sb500366v 25871405 10.1021/sb500366v

[22] Obst U, Lu TK, Sieber V. A modular toolkit for generating Pichia pastoris secretion libraries. ACS Synth Biol. 2017; acssynbio.6b00337. doi: 10.1021/acssynbio.6b00337 28252957 10.1021/acssynbio.6b00337

[23] Schindler D, Milbredt S, Sperlea T, Waldminghaus T. Design and assembly of DNA sequence libraries for chromosomal insertion in bacteria based on a set of modified MoClo vectors. ACS Synth Biol. 2016; acssynbio.6b00089. doi: 10.1021/acssynbio.6b00089 27306697 10.1021/acssynbio.6b00089

[24] Moore SJ, Lai HE, Kelwick RJR, Chee SM, Bell DJ, Polizzi KM, et al EcoFlex: A Multifunctional MoClo Kit for E. coli Synthetic Biology. ACS Synth Biol. 2016;5: 1059–1069. doi: 10.1021/acssynbio.6b00031 27096716 10.1021/acssynbio.6b00031

[25] Iverson S V., Haddock TL, Beal J, Densmore DM. CIDAR MoClo: Improved MoClo Assembly Standard and New E. coli Part Library Enable Rapid Combinatorial Design for Synthetic and Traditional Biology. ACS Synth Biol. 2016;5: 99–103. doi: 10.1021/acssynbio.5b00124 26479688 10.1021/acssynbio.5b00124

[26] Alieva NO, Konzen K a., Field SF, Meleshkevitch E a., Hunt ME, Beltran-Ramirez V, et al Diversity and evolution of coral fluorescent proteins. PLoS One. 2008;3: 1–12. doi: 10.1371/journal.pone.0002680 18648549 10.1371/journal.pone.0002680 PMC2481297

[27] Patron N, et al Standards for Plant Synthetic Biology: A Common Syntax for Exchange of DNA Parts. New Phytol. 2015; 13–19. 10.1111/nph.13532 26171760

[28] Pedelacq J-D, Cabantous S, Tran T, Terwilliger TC, Waldo GS. Engineering and characterization of a superfolder green fluorescent protein. Nat Biotech. Nature Publishing Group; 2006;24: 79–88. Available: http://dx.doi.org/10.1038/nbt1172 10.1038/nbt1172 16369541

[29] August PR, Grossman TH, Minor C, Draper MP, Macneil IA, Pemberton JM, et al Biosynthetic Pathway from Chromobacterium violaceum 513 Sequence Analysis and Functional Characterization of the Violacein Biosynthetic Pathway from Chromobacterium violaceum JMMB Research Article. J Mol Microbiol Biotechnol. 2000;2: 513–519. 11075927

[30] Misawa N, Shimada H. Metabolic engineering for the production of carotenoids in non- carotenogenic bacteria and yeasts. J Biotechnol. 1998;59: 169–181. doi: 10.1016/S0168-1656(97)00154-5 10.1016/s0168-1656(97)00154-5 9519479

[31] Misawa N, Nakagawa M, Kobayashi K, Yamano S, Izawa Y, Nakamura K, et al Elucidation of the Erwinia uredovora carotenoid biosynthetic pathway by functional analysis of gene products expressed in Escherichia coli. J Bacteriol. Central Laboratories for Key Technology, Kirin Brewery Co., Ltd., Gunma, Japan.; 1990;172: 6704–6712. 10.1128/jb.172.12.6704-6712.1990 PMC210783 2254247

[32] Nishizaki T, Tsuge K, Itaya M, Doi N, Yanagawa H. Metabolic engineering of carotenoid biosynthesis in Escherichia coli by ordered gene assembly in Bacillus subtilis. Appl Environ Microbiol. 2007;73: 1355–1361. doi: 10.1128/AEM.02268-06 17194842 10.1128/AEM.02268-06 PMC1828653

[33] Rodriguez D. A Guide to Carotenoid Analysis in Foods. Life Sciences. 2001.

[34] Ceroni F, Algar R, Stan G-B, Ellis T. Quantifying cellular capacity identifies gene expression designs with reduced burden. Nat Methods. 2015;12: 1–8. doi: 10.1038/nmeth.3339 25849635 10.1038/nmeth.3339

[35] Sambrook J, Russell DW. The Inoue Method for Preparation and Transformation of Competent E. Coli: “Ultra-Competent” Cells. Cold Spring Harb Protoc. 2006;2006: pdb.prot3944. doi: 10.1101/pdb.prot3944 32482900 10.1101/pdb.prot101196

