# Supplementary material for: Mobius Assembly: A versatile Golden-Gate framework towards universal DNA assembly
Source: PLoS One. 2018 Jan 2;13(1):e0189892. doi: 10.1371/journal.pone.0189892 (PMC5749717; doi:10.1371/journal.pone.0189892)
Supplement: S1 Fig — Level 2 cloning was optimized for the different reaction buffers and DNA ligases. The chromoprotein TUs tsPurple, amilGFP, asCP, and aeBlue were combined in Level 2 Acceptor Vectors, and the cells with successfully assembled constructs grew into blue colonies. Different sets of reagents tested were: (A) Buffer AarI + T4 DNA ligase, (B) 2x Tango Buffer + T4 DNA ligase, (C) T4 DNA Ligase Buffer + T4 DNA ligase, (D) Buffer AarI + T7 DNA ligase, (E) 2x Tango Buffer T7 DNA ligase, (F) T4 DNA Ligase Buffer + T7 DNA ligase. T4 DNA Ligase Buffer + T4 DNA ligase combination resulted in the optimum assembly efficiency. (PDF) [file pone.0189892.s001.pdf]

As the protocol for Golden Gate Assembly with *Aar*I was not optimized before, two sets of experiments were conducted to optimize the efficiency of Level 2 assembly (four TUs plus one linker). Optimization was conducted for different buffers and DNA ligases. More specifically *Aar*I unique buffer, 2x Tango Buffer (recommended for double digests by the supplier of *Aar*I), and T4 DNA ligase buffer were tested in combination with T4 DNA ligase (Thermo Fisher Scientific) or T7 DNA ligase (NEB). The different sets of DNA ligase and buffer were tested for Level 2 assembly of four chromoprotein TUs (*tsPurple*, *amilGFP*, *asCP*, and *aeBlue* genes, each with J23103 promoter and T7Te terminator). The assembly efficiency was significantly higher when the reaction was carried out in T4 DNA ligase buffer with T4 DNA ligase (Supporting Figure 1 and Table 1), and thus we have chosen that combination for our construction.

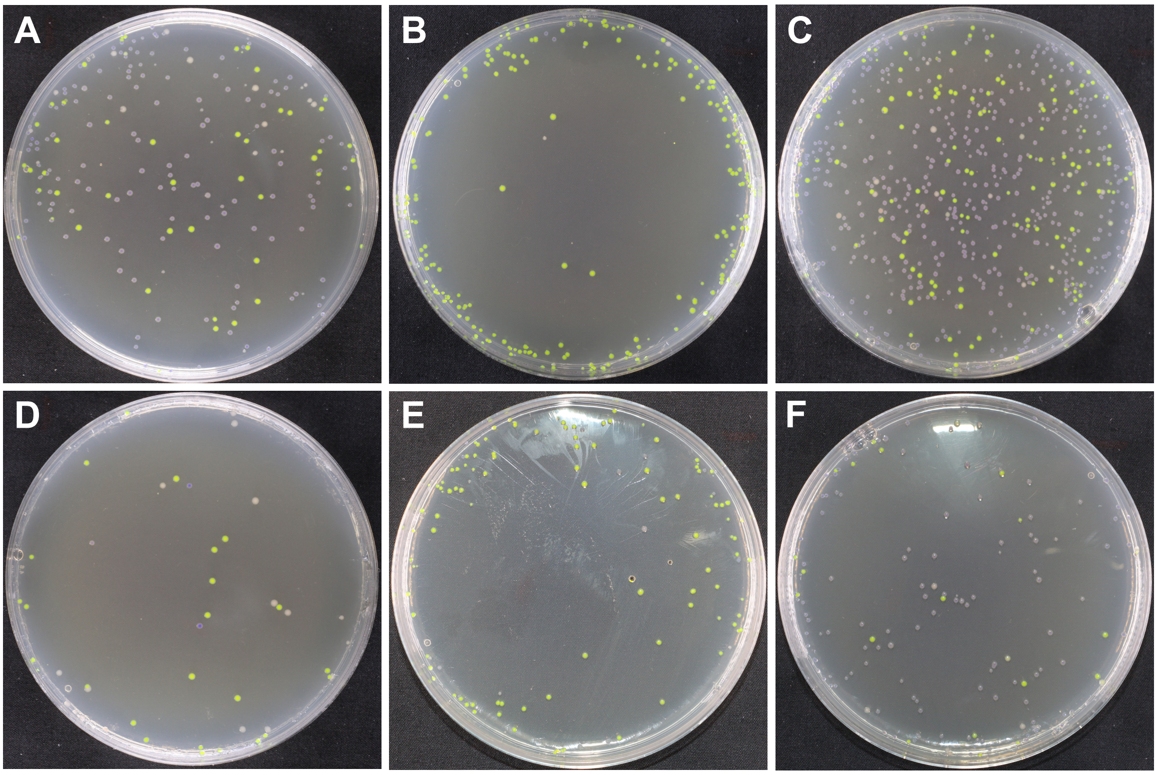

Supporting Figure 1. Optimization of Level 2 assembly reagents.
Level 2 cloning was optimized for the different reaction buffers and DNA ligases. The chromoprotein TUs *tsPurple, amilGFP, asCP, and aeBlue* were combined in Level 2 Acceptor Vectors, and the cells with successfully assembled constructs grew into blue colonies. Different sets of reagents tested were: (A) Buffer *Aar*I + T4 DNA ligase, (B) 2x Tango Buffer + T4 DNA ligase, (C) T4 DNA Ligase Buffer + T4 DNA ligase, (D) Buffer *Aar*I + T7 DNA ligase, (E) 2x Tango Buffer T7 DNA ligase, (F) T4 DNA Ligase Buffer + T7 DNA ligase. T4 DNA Ligase Buffer + T4 DNA ligase combination resulted in the optimum assembly efficiency.
