# Supplementary material for: Mobius Assembly: A versatile Golden-Gate framework towards universal DNA assembly
Source: PLoS One. 2018 Jan 2;13(1):e0189892. doi: 10.1371/journal.pone.0189892 (PMC5749717; doi:10.1371/journal.pone.0189892)
Supplement: S2 Fig — Level 2 cloning was optimized for the different thermocycling conditions used for the assembly reactions. The chromoprotein TUs tsPurple, eforRed, asCP and mRFP1 were cloned into Level 2 Acceptor Vectors under different thermocycling conditions (digestion/ligation). Cells with successfully assembled constructs grew into pink colonies. (PDF) [file pone.0189892.s002.pdf]

Furthermore, we tested thermocycling conditions with varied digestion and ligation times for Level 2 assembly. Assembly of the four red-class chromoprotein TUs (*tsPurple*, *efforRed*, *asCP*, or *mRFP1* gene together with J23103 promoter and T7Te terminator) were used to test the effect of the thermocycling conditions on the assembly efficiency. Six different thermocycling conditions were tested, all of which were identical in total duration (1 hour and 30min) but varying the period of restriction digestion and ligation, the ratio between them, and the number of the cycles of digestion/ligation. No considerable difference was observed among the various thermocycling conditions for the assembly efficiency of the 3.2kb 4TU construct (Supporting Figure 2). Assembly of much larger constructs, however, might be more efficient if many cycles of short digestion/ligation are implemented. The assembly efficiency dropped across different conditions when old reagents (e.g. four months since opening) were used (Supporting Table 2).

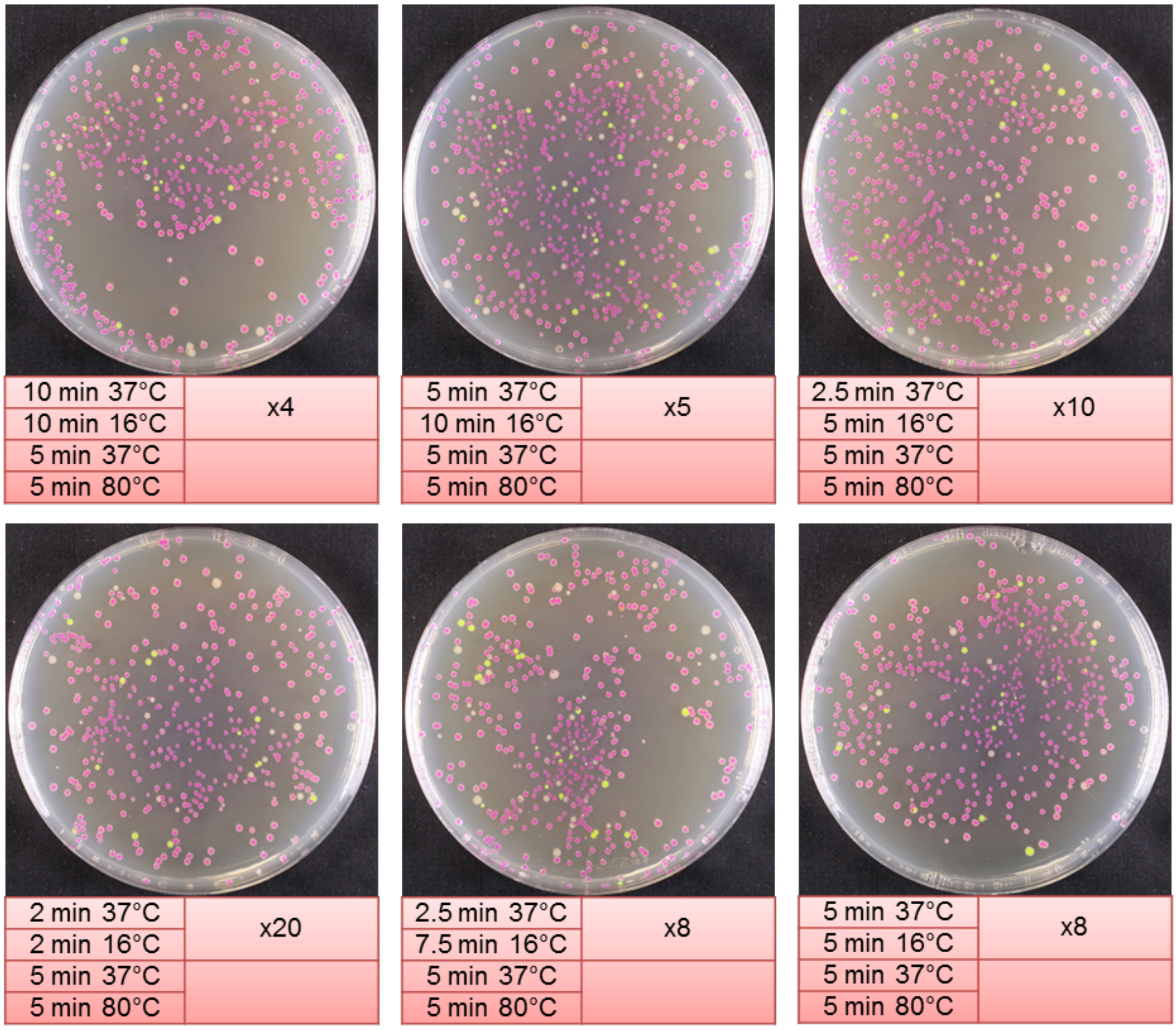

Supporting Figure 2. Optimization of Level 2 assembly reaction conditions.
Level 2 cloning was optimized for the different thermocycling conditions used for the assembly reactions. The chromoprotein TUs *tsPurple*, *eforRed*, *asCP* and *mRFP1* were cloned into Level 2 Acceptor Vectors under different thermocycling conditions (digestion/ligation). Cells with successfully assembled constructs grew into pink colonies.
