# Supplementary material for: Mobius Assembly: A versatile Golden-Gate framework towards universal DNA assembly
Source: PLoS One. 2018 Jan 2;13(1):e0189892. doi: 10.1371/journal.pone.0189892 (PMC5749717; doi:10.1371/journal.pone.0189892)
Supplement: S1 Table — (PDF) [file pone.0189892.s003.pdf]

Supporting Table 1. Colony counts for the Level 2 assembly reagents.

	Buffer-ligase combinations					
	T4 DNA Ligase Buffer- T4 DNA ligase	T4 DNA Ligase Buffer- T7 DNA ligase	2xTango Buffer- T4 DNA ligase	2xTango Buffer- T7 DNA ligase	AarI Buffer- T4 DNA ligase	AarI Buffer- T7 DNA ligase
Replicate 1						
# blue colonies	**159**	46	7	3	46	4
# yellow colonies	31	12	49	13	4	3
# white colonies	0	1	0	0	0	0
Replicate 2						
# blue colonies	**484**	86	5	8	124	10
# yellow colonies	117	15	124	75	43	18
# white colonies	8	3	2	1	9	4
Replicate 3						
# blue colonies	**298**	162	21	16	54	21
# yellow colonies	90	40	167	268	372	192
# white colonies	8	13	4	3	2	3
