# Supplementary material for: Mobius Assembly: A versatile Golden-Gate framework towards universal DNA assembly
Source: PLoS One. 2018 Jan 2;13(1):e0189892. doi: 10.1371/journal.pone.0189892 (PMC5749717; doi:10.1371/journal.pone.0189892)
Supplement: S2 Table — (PDF) [file pone.0189892.s004.pdf]

Supporting Table 2. Colony counts for the Level 2 assembly optimization.

	Replicate 1					
Digestion (min)-Ligation (min)	**10 - 10**	**5 - 10**	**2.5 - 5**	**2 - 2**	**2.5 - 7.5**	**5 - 5**
# pink colonies	268	438	323	279	289	368
# yellow colonies	9	28	14	9	26	13
# white colonies	13	20	13	9	15	16
Pink/total colonies	0.92	0.90	0.92	0.94	0.88	0.93
Yellow/total colonies	0.03	0.06	0.04	0.03	0.08	0.03
White/total colonies	0.04	0.04	0.04	0.03	0.05	0.04

	Replicate 2					
Digestion (min)-Ligation (min)	**10 - 10**	**5 - 10**	**2.5 - 5**	**2 - 2**	**2.5 - 7.5**	**5 - 5**
# pink colonies	179	104	254	184	171	163
# yellow colonies	22	13	30	22	20	19
# white colonies	1	1	3	2	2	2
Pink/total colonies	0.89	0.88	0.89	0.88	0.89	0.89
Yellow/total colonies	0.11	0.11	0.10	0.11	0.10	0.10
White/total colonies	0.00	0.01	0.01	0.01	0.01	0.01

	Replicate 3					
Digestion (min)-Ligation (min)	**10 - 10**	**5 - 10**	**2.5 - 5**	**2 - 2**	**2.5 - 7.5**	**5 - 5**
# pink colonies	188	135	140	202	186	264
# yellow colonies	64	55	43	81	78	60
# white colonies	4	3	5	3	3	3
Pink/total colonies	0.73	0.70	0.74	0.71	0.70	0.81
Yellow/total colonies	0.25	0.28	0.23	0.28	0.29	0.18
White/total colonies	0.02	0.02	0.03	0.01	0.01	0.01
