# Supplementary material for: Mobius Assembly: A versatile Golden-Gate framework towards universal DNA assembly
Source: PLoS One. 2018 Jan 2;13(1):e0189892. doi: 10.1371/journal.pone.0189892 (PMC5749717; doi:10.1371/journal.pone.0189892)
Supplement: S3 Table — (PDF) [file pone.0189892.s005.pdf]

Supporting Table 3. Vector toolkit: features and database and repository IDs

Vector name	GenBank Accession numbers	Addgene Plasmid ID	Origin of replication	Antibiotic Resistance	Negative selection marker	Function
mUAV	MG252981	102680	pMB1	Chloramphenicol	amilCP	Conversion of a DNA fragment into a standard part
pMA_Level1A	MG252982	102701	pMB1	Kanamycin	spisPink	Level 1 cloning: Assembly of standard parts into a TU or augmentation of high-order multi-TUs
pMA_Level1B	MG252983	102702	pMB1	Kanamycin	spisPink	
pMA_Level1Γ	MG252984	102703	pMB1	Kanamycin	spisPink	
pMA_Level1Δ	MG252985	102704	pMB1	Kanamycin	spisPink	
pMA_Level1A	MG252986	102705	pMB1	Chloramphenicol	sfGFP	Level 2 cloning: Assembly of multiple TUs into a multi-TU construct or augmentation of higher order multi-TUs
pMA_Level1B	MG252987	102706	pMB1	Chloramphenicol	sfGFP	
pMA_Level1Γ	MG252988	102707	pMB1	Chloramphenicol	sfGFP	
pMA_Level1Δ	MG252989	102708	pMB1	Chloramphenicol	sfGFP	
pMA_Auxiliray_1	MG252990	102709	pMB1	Kanamycin	-	Axillary plasmids to provide overhangs in the Level 2 cloning
pMA_Auxiliray_2	MG252991	102710	pMB1	Kanamycin	-	
pMA_Auxiliray_3	MG252992	102711	pMB1	Kanamycin	-	
pMA_Auxiliray_4A	MG252993	102712	pMB1	Kanamycin	-	
pMA_Auxiliray_4B	MG252994	102713	pMB1	Kanamycin	-	
pMA_Auxiliray_4Γ	MG252995	102714	pMB1	Kanamycin	-	
pMA_Auxiliray_4Δ	MG252996	102715	pMB1	Kanamycin	-	
